# Diagnostic Validity of the Generalized Anxiety Disorder - 7 (GAD-7) among Pregnant Women

**DOI:** 10.1371/journal.pone.0125096

**Published:** 2015-04-27

**Authors:** Qiu-Yue Zhong, Bizu Gelaye, Alan M. Zaslavsky, Jesse R. Fann, Marta B. Rondon, Sixto E. Sánchez, Michelle A. Williams

**Affiliations:** 1 Department of Epidemiology, Harvard T. H. Chan School of Public Health, Boston, MA, United States of America; 2 Department of Health Care Policy, Harvard Medical School, Boston, MA, United States of America; 3 Department of Psychiatry and Behavioral Sciences, University of Washington, Seattle, WA, United States of America; 4 Department of Medicine, Cayetano Heredia Peruvian University, Lima, Peru; 5 Universidad Peruana de Ciencias Aplicadas, Lima, Peru; 6 Asociación Civil PROESA, Lima, Peru; Harvard Medical School, UNITED STATES

## Abstract

**Objective:**

Generalized anxiety disorder (GAD) during pregnancy is associated with several adverse maternal and perinatal outcomes. A reliable and valid screening tool for GAD should lead to earlier detection and treatment. Among pregnant Peruvian women, a brief screening tool, the GAD-7, has not been validated. This study aims to evaluate the reliability and validity of the GAD-7.

**Methods:**

Of 2,978 women who attended their first perinatal care visit and had the GAD-7 screening, 946 had a Composite International Diagnostic Interview (CIDI). The Cronbach’s alpha was calculated to examine the reliability. We assessed the criterion validity by calculating operating characteristics. The construct validity was evaluated using factor analysis and association with health status on the CIDI. The cross-cultural validity was explored using the Rasch Rating Scale Model (RSM).

**Results:**

The reliability of the GAD-7 was good (Cronbach’s alpha = 0.89). A cutoff score of 7 or higher, maximizing the Youden Index, yielded a sensitivity of 73.3% and a specificity of 67.3%. One-factor structure of the GAD-7 was confirmed by exploratory and confirmatory factor analysis. Concurrent validity was supported by the evidence that higher GAD-7 scores were associated with poor self-rated physical and mental health. The Rasch RSM further confirmed the cross-cultural validity of the GAD-7.

**Conclusion:**

The results suggest that the Spanish-language version of the GAD-7 may be used as a screening tool for pregnant Peruvian women. The GAD-7 has good reliability, factorial validity, and concurrent validity. The optimal cutoff score obtained by maximizing the Youden Index should be considered cautiously; women who screened positive may require further investigation to confirm GAD diagnosis.

## Introduction

Characterized by excessive anxiety and worry about everyday events or activities [1], generalized anxiety disorder (GAD) is one of the most common mental disorders [2]. GAD disproportionally affects women, especially those during childbearing age [3]. Maternal anxiety during pregnancy is associated with several adverse outcomes including spontaneous abortion, preeclampsia, placenta abruption, preterm labor, low birth weight, smaller head circumference, and lower mental developmental scores in infants [4–9]. Well-established literature has shown that women who experience anxiety disorder during pregnancy are at higher risk of postpartum depression and comorbid anxiety [10]. Unfortunately, identifying GAD is challenging. GAD has the lowest diagnostic reliability among any anxiety disorders and is often neglected by obstetricians [11, 12]. To effectively diagnose and treat GAD during pregnancy, early detection, which requires the use of reliable and valid screening tools, is crucial [13].

The Generalized Anxiety Disorder-7 (GAD-7) is a 7-item questionnaire for GAD exploring the 2-week period prior to screening [2]. Globally, among clinical and general population samples, the GAD-7 has demonstrated good reliability and cross-cultural validity as a measure of GAD [14]. However, the GAD-7 has not yet been validated among pregnant women in low-middle income countries (LMICs) including Peru, where GAD and comorbid depression are among the leading causes of morbidity and mortality [15]. A recent study by de Paz et al. [5] found that 25% of pregnant Peruvian women reported mild to severe anxiety symptoms using the Depression Anxiety Stress Scales (DASS-21).

Given that there is no validation for the GAD-7 among pregnant Peruvian women, we seek to evaluate the reliability and diagnostic validity of the Spanish-language version of the GAD-7 for detecting antepartum GAD using the Composite International Diagnostic Interview (CIDI) as the gold standard. Utilizing classic test theory, our primary aim is to evaluate the reliability, criterion validity, and construct validity including factorial and concurrent validity of the GAD-7. Our secondary aim is to evaluate the validity of the GAD-7 using the Rasch Rating Scale Model (RSM).

## Methods

All participants provided written informed consent. The institutional review boards of the Instituto Nacional Materno Perinatal, Lima, Peru and the Harvard T.H. Chan School of Public Health Office of Human Research Administration, Boston, MA approved all procedures used in this study.

### Study population

This cross-sectional study was a part of the Pregnancy Outcomes, Maternal and Infant Study (PrOMIS) Cohort, which is an ongoing prospective cohort study of pregnant women enrolled in prenatal care clinics at the Instituto Nacional Materno Perinatal (INMP) in Lima, Peru. Under the aegis of the Peruvian Ministry of Health, the INMP is the primary referral hospital for maternal and perinatal care. From February 2012 to March 2014, starting with the first prenatal care visit, women who attended the INMP were recruited for this investigation. Pregnant women, 18–49 years, with a gestational age ≤ 16 weeks and who spoke and understood Spanish were eligible for inclusion.

### Data collection

Of the 3,775 eligible participants, 3,045 underwent a structured in-person interview. The structured interview collected information regarding maternal socio-demographics, lifestyle characteristics, medical and reproductive history, abuse history, and GAD symptoms. Due to missing information on the GAD-7, 67 women were excluded, leaving 2,978 women with completed GAD-7 information in this analysis.

Due to cost and time restrictions, a subset of participants (41.7%, *n* = 1,271) was randomly selected for the diagnostic interview within 15 days of the initial structured interview. Of the 1,271 women selected, 956 completed the diagnostic interview. A total of 315 women did not participate in the diagnostic interviews for the following reasons: 123 women were not reached within the stipulated 14 days after screening; 96 women were no longer eligible due to abortions, malformation or twin pregnancies; 56 women were excluded due to change of address or inaccurate contact information; and 40 women refused to participate citing reasons such as lack of time. Of the 956 women, 10 women missing information on the GAD-7 were excluded. Subsequently, 946 women with completed GAD-7 and diagnostic interview information remained in the current analysis.

### Scales

#### Generalized Anxiety Disorder—7

The GAD-7 is a 7-item questionnaire developed to identify probable cases of GAD and measure the severity of GAD symptoms [2]. The GAD-7 assesses the most prominent diagnostic features (diagnostic criteria A, B, and C from the *Diagnostic and Statistical Manual of Mental Disorders*, fourth edition [DSM-IV]) for GAD [14, 16]. The GAD-7 items include: 1) nervousness; 2) inability to stop worrying; 3) excessive worry; 4) restlessness; 5) difficulty in relaxing; 6) easy irritation; and 7) fear of something awful happening. The GAD-7 asks participants to rate how often they have been bothered by each of these 7 core symptoms over the past 2 weeks. Response categories are “not at all,” “several days,” “more than half the days,” and “nearly every day,” scored as 0, 1, 2, and 3, respectively. The total score of the GAD-7 ranges from 0 to 21. Among primary care patients and the general population, the GAD-7 has demonstrated good internal consistency, test-retest reliability, and convergent, construct, criterion, and factorial validity [2, 14, 17, 18]. In the original validation study performed in the primary care clinics [2], the cutoff score of 10 or higher (recommended cutoff score) provides a sensitivity of 89% and a specificity of 82%.

#### The World Health Organization World Mental Health Composite International Diagnostic Interview

The World Health Organization World Mental Health Composite International Diagnostic Interview (WHO WMH-CIDI) (hereafter referred to as CIDI) is a comprehensive, fully structured interview designed for the assessment of mental disorders according to the criteria of the International Classification of Diseases-10 (ICD-10) and the DSM-IV [19]. Of note, the CIDI has not yet been updated using DSM-5. The CIDI is a reliable, valid, and practical instrument which can be used cross-culturally [19–22]. The lifetime, 12-month, and 30-day diagnosis of GAD has been generated based on both the ICD-10 and the DSM-IV. In this analysis, we used the DSM-IV diagnosis for 12-month prevalence as the gold standard because cases with GAD episodes for < 6 months did not differ greatly from those ≥ 6 months [2, 23]. Four licensed research psychologists were recruited and received structured training on administration of the CIDI. The training program was similar to the one that one of the co-authors (BG) had attended at the Social Survey Institute at the University of Michigan (WHO Training Center). In addition to the structured training course for the interviewers, item-by-item description of questionnaires and role-plays were used. To ensure highest quality of data collection, while interviewers were in the field, they were provided strict on-site supervision and support. All paper and pencil recorded questionnaires collected manually were entered using Blaise version 4.6 (Statistics Netherlands), which contained the entire WMH-CIDI algorithm along with an automatic checking mechanism to identify item omissions and unusual responses.

### Statistical analysis

#### Reliability

We assessed the reliability using several agreement and consistency indices. Specifically, the Cronbach’s alpha was computed to assess the internal consistency for the GAD-7.

#### Validity

The criterion validity for the GAD-7 was assessed based on the CIDI diagnosis of GAD. We computed the following operating characteristics: sensitivity, specificity, positive likelihood ratio (LR+), negative likelihood ratio (LR-), positive predictive values (PPV), and negative predictive values (NPV). Additionally, to identify the best cutoff score for GAD among pregnant Peruvian women, the Youden Index was calculated as a metric for the cutoff decision [24]. The Youden Index is defined as *J* = max_*c*_{*Sensitivity*(*c*)+*Specificity*(*c*)-1} and ranges from 0 to 1 [25]. The receiver operating characteristic (ROC) curve analysis was used to identify the optimal balance of sensitivity and specificity and the area under the ROC curve (AUC).

A subset of women screened with the GAD-7 was selected for CIDI diagnostic interviews. Considering the possibility of verification bias, the Begg and Greens adjusted estimates for operating characteristics and 95% confidence intervals (CIs) were calculated to correct for this bias [26, 27].

Using the exploratory factor analysis (EFA) and the confirmatory factor analysis (CFA), the factor structure of the GAD-7 was explored. The suitability for performing the factor analysis was assessed prior to undertaking the factor analysis. The result of the suitability analysis supported the appropriateness of proceeding with the factor analysis (Bartlett’s test of sphericity, *P* < 0.001; the Kaiser-Meyer-Olkin measure of sampling adequacy = 0.91). Then, the EFA was conducted using the maximum likelihood (ML) method. The scree plot and eigenvalues associated with each factor were used to identify the number of meaningful factors. Factors with relatively large eigenvalues (> 1) were assumed to be meaningful and retained for rotation [28]. Factor loadings ≥ 0.4 were used in the factor designation.

To complement the EFA and evaluate the fit of the one-factor model identified in the literature [2], we conducted the CFA. Due to violation of the multivariate normality assumption, the weighted least squares (WLS) estimation was adopted. The standardized root mean square residual (SRMR), the comparative fit index (CFI), and the root mean square error of approximation (RMSEA) along with 90% confidence intervals (90% CIs) were calculated to evaluate model fit [29]. Brown [29] recommended that the following criteria provided evidence for reasonably good fit: 1) SRMR close to 0.08 or below; 2) CFI close to 0.95 or above; and 3) RMSEA close to 0.06 or below.

Prior research has shown that anxiety is associated with poor or reduced functional status [17]. We hypothesized that higher GAD-7 scores were associated with poor self-rated physical and mental health status. Using 2 screening questions from the CIDI, which asked participants to rate overall physical and mental health, the construct validity of the GAD-7 was evaluated. The chi-square test was used to compare the proportions of self-rated, fair and poor physical and mental health between participants classified as GAD and non-GAD according to the GAD-7.

#### Item Response Theory Models

To evaluate the GAD-7, we first applied the Rasch RSM, an item-based approach where ordinal observed item scores were transformed to linear measures representing the underlying latent construct [30–32]. This model was based on a mathematical model where the probability of endorsing an item was a logistic function of the difference between the person’s level of anxiety and the level of anxiety expressed by the item (item difficulty) [30, 33–35]. Under the Rasch RSM, a single set of mean response thresholds was estimated, and the discrimination was assumed the same for all 7 items [31, 32]. Considering controversy regarding disordered thresholds, we first completed the Rasch RSM analysis using the method proposed by Forkmann [35], and fully described in our previous publication [36]. In particular, in the case of disordered thresholds (an indicator of disordered response categories), Forkmann et al. suggested collapsing adjacent categories to improve fit [30, 31, 35, 37, 38]. However, Adams et al. [39] argued that regardless of the order of the thresholds, the response categories were ordered when the data fit the Rasch model; disordered thresholds were not necessarily a problem. The disordered thresholds were indicative of low frequencies in some response categories. To illustrate Adams’ argument, the frequency and average ability of participants endorsing each response category were also examined.

For additional analysis, we further explored the discrimination of the GAD-7 items using a more flexible Item Response Theory (IRT) model, the Generalized Partial Credit Model (GPCM) [40]. Discrimination parameters described the item’s ability to discriminate between persons with different underlying GAD status [41]. The ability to differentiate women’s anxiety levels for an item with a low discrimination parameter was lower than that of an item with a higher discrimination parameter. Discrimination parameters > 0.64 reflected a moderate discrimination [42, 43].

Statistical analyses were performed using SAS 9.3 (SAS Institute, Cary, NC, USA), Stata 11.0 (Statacorp, College Station, TX), Winsteps 3.80.0 (Chicago, Illinois), and R 3.1.0 using the “irt” package. The level of statistical significance was set at *P*-value < 0.05 and all tests were two-sided.

## Results

### Participant characteristics

A summary of selected socio-demographic and reproductive characteristics of study participants is presented in Table 1. In total, 2,978 participants between 18 and 48 years (mean age = 28.0 years; standard deviation, SD = 6.2 years) were included. The majority of the participants were Mestizo (75.1%) and married or living with a partner (80.9%) with at least 7 years of education (95.5%). In this study, 46.0% of the participants were employed and 50.3% reported having difficulty in paying for basic foods. Two-thirds (66.6%) of the participants rated health as poor during current pregnancy. The average gestational age at interview was 9.6 (SD = 3.4) weeks. Between women with completed diagnostic interview information and women with the GAD-7 screening only (without the CIDI diagnostic interview), no significant difference regarding above characteristics was observed (Table 1).

**Table 1:** Socio-demographics and Reproductive Characteristics of Entire Study Population (N = 2,978), Women Participating Diagnostic Interview (n = 946), and Women with the Generalized Anxiety Disorder-7 (GAD-7) Screening only (n = 2,032).

Characteristics	All (N = 2,978)		Diagnostic interview (N = 946)		GAD-7 screening only (N = 2,032)		*P-*value**
	n	%	n	%	n	%	
Maternal age (years)*	28.0 ± 6.2		28.2 ± 6.2		28.1 ± 6.4		0.55
Maternal age (years)							
18–20	160	5.4	46	4.9	114	5.6	0.82
20–29	1662	55.8	533	56.3	1129	55.6	
30–34	620	20.8	200	21.1	420	20.7	
≥35	536	18.0	167	17.7	369	18.2	
Education (years)							
≤6	125	4.2	39	4.1	86	4.2	0.99
7–12	1633	54.8	519	54.9	1114	54.8	
>12	1213	40.7	387	40.9	826	40.7	
Mestizo	2237	75.1	728	77.0	1509	74.3	0.14
Married/living with partner	2409	80.9	755	79.8	1654	81.4	0.31
Employed during pregnancy	1371	46.0	442	46.7	929	45.7	0.61
Access to basic foods							
Hard	1496	50.3	469	49.6	1027	50.5	0.61
Not very hard	1480	49.7	477	50.4	1003	49.4	
Self-reported health status during pregnancy							
Good	920	30.9	287	30.3	633	31.2	0.61
Poor	1984	66.6	639	67.6	1345	66.2	
Gestational age at interview*	9.6 ± 3.4		9.8 ± 4.1		9.6 ± 4.1		0.11

Due to missing data, percentages may not add up to 100%.

*mean ± SD (standard deviation)

** *P*-value was calculated using the Chi-square test or the Fisher’s exact test for categorical variables. *P-*value was calculated using the Wilcoxon rank sum test for continuous variables.

Distributions of socio-demographic and reproductive characteristics according to women’s GAD status, as defined by the CIDI, are presented in Table 2. Fourteen women fulfilled the DSM-IV criteria for GAD over the past 12 months. Compared with women without GAD diagnosis, women with GAD diagnosis were less likely to be in the age range of 20–29 years and more likely to have difficulty paying for basic foods. Additionally, women with GAD diagnosis had statistically significantly higher mean GAD-7 scores than women without GAD diagnosis (mean = 9.9, SD = 5.7 vs. mean = 5.7, SD = 4.9; *P-*value = 0.002).

**Table 2:** Socio-demographics and Reproductive Characteristics of Study Population by the Composite International Diagnostic Interview (CIDI) Diagnosed Generalized Anxiety Disorder Status (N = 946).

Characteristics	Participants (N = 946)				
	Anxiety (n = 14)		No anxiety (n = 932)		*P-*value**
	n	%	n	%	
Maternal age*	29.1 ± 5.6		28.2 ± 6.2		0.41
Maternal age (years)					
18–20	2	14.3	44	4.7	**0.03**
20–29	4	28.6	529	56.8	
30–34	6	42.9	194	20.8	
≥35	2	14.3	165	17.7	
Education (years)					
≤6	1	7.1	38	4.1	0.81
7–12	8	57.1	511	54.8	
>12	5	35.7	382	41.0	
Mestizo	11	78.6	717	76.9	1.00
Employed during pregnancy	4	28.6	438	47.0	0.19
Married/living with a partner	10	71.4	745	79.9	0.50
Access to basic foods					
Hard	12	85.8	457	49.1	**<0.01**
Not very hard	2	14.3	475	51.0	
Self-reported health status during pregnancy					
Good	3	21.4	284	30.5	0.76
Poor	10	71.4	629	67.5	
Gestational age at interview*	9.7 ± 2.6		9.8 ± 4.1		0.98
GAD-7 score*	9.9 ± 5.7		5.7 ± 4.9		**0.002**

Due to missing data, percentages may not add up to 100%.

*mean ± SD (standard deviation)

** *P*-value was calculated using the Chi-square test or the Fisher’s exact test for categorical variables. *P-*value was calculated using the Wilcoxon rank sum test for continuous variables.

### Reliability

The internal consistency of the GAD-7 gave a Cronbach’s alpha of 0.89. The correlations between the 7 items of the GAD-7 and the total scores ranged from 0.61 to 0.73 (*P*-value < 0.0001) (Table 3).

**Table 3:** Item-total Correlation, Alpha if Item deleted, and Factor Loading of the Generalized Anxiety Disorder-7 (GAD-7).

Item	Corrected item-total correlation	Alpha if item deleted	Factor loading
1. Feeling nervous, anxious, or on edge	0.68	0.87	0.73
2. Not being able to stop or control worrying	0.72	0.86	0.78
3. Worrying too much about different things	0.73	0.86	0.80
4. Trouble relaxing	0.69	0.87	0.74
5. Being so restless that it’s hard to sit still	0.70	0.87	0.74
6. Becoming easily annoyed or irritable	0.61	0.88	0.64
7. Feeling afraid as if something awful might happen	0.61	0.88	0.64
GAD-7 sum score	N/A	0.89*	N/A

*Overall Cronbach’s alpha

### Validity

#### Criterion Validity

**Fig 1:**
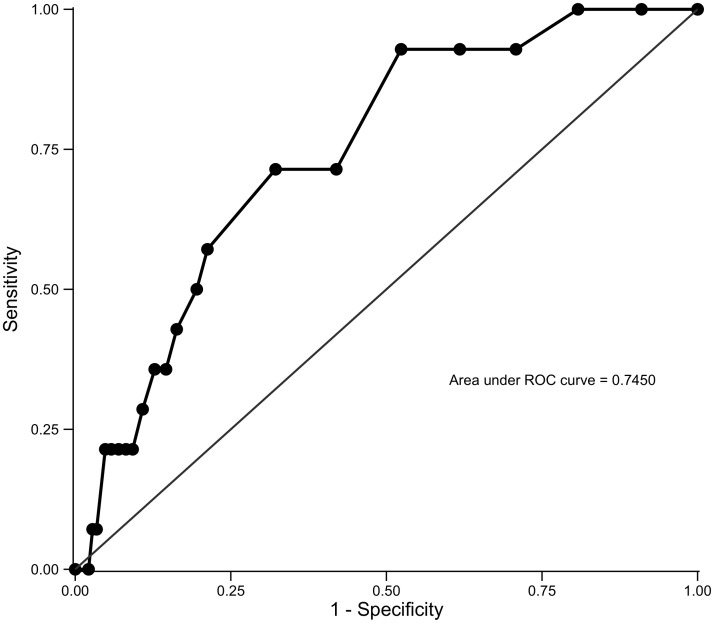
Receiver Operating Characteristics (ROC) Curves of Generalized Anxiety Disorder 7-Item (GAD-7) Scores.

Using the CIDI DSM-IV 12-month GAD diagnosis as the gold standard, Table 4 summarizes the operating characteristics of the GAD-7. The optimal cutoff score to maximize the Youden Index was a score ≥ 7. At this score, the sensitivity and specificity were 73.3% (95% CI: 58.1%- 85.4%) and 67.3% (95% CI: 65.5%- 69.0%), respectively; the LR+ was 2.2 (95% CI: 1.9–2.7) and LR- was 0.4 (95% CI: 0.2–0.6). Women with GAD were 2.2 times more likely than women without GAD to have a GAD-7 score ≥ 7. A LR- of 0.4 indicated that women with GAD were 0.4 times as likely as women without GAD to have a GAD-7 score < 7. The PPV was 3.3% (95% CI: 2.3%- 4.6%) and NPV was 99.4% (95% CI: 98.9%- 99.7%) (Table 4, S1 Table). The AUC under the ROC curve for detecting GAD was 0.75 (95% CI: 0.68–0.80) with a standard error of 0.03 (Fig 1).

**Table 4:** Begg and Greens Adjusted Sensitivity and Specificity for Generalized Anxiety Disorder Diagnosis across Various Cutoff Scores of the Generalized Anxiety Disorder-7 (GAD-7).

Cutoff scores	Sensitivity (95%CI)	# True positive	Specificity (95%CI)	# True negative	Youden index	LR+ (95% CI)	LR- (95%CI)	PPV (95%CI)	NPV (95%CI)	Prevalence
**Score ≥5**	93.3 (81.7, 98.6)	42	46.3 (44.5, 48.2)	1375	39.6	1.7 (1.6, 1.9)	0.1 (0.1, 0.4)	2.6 (1.9, 3.5)	99.8 (99.4, 100.0)	53.8
**Score ≥6**	73.3 (58.1, 85.4)	33	56.1 (54.3, 57.9)	1646	29.4	1.7 (1.4, 2.0)	0.5 (0.3, 0.8)	2.5 (1.7, 3.5)	99.3 (98.7, 99.6)	44.4
**Score ≥7**	**73.3 (58.1, 85.4)**	**32**	**67.3 (65.5, 69.0)**	**1974**	**40.6**	**2.2 (1.9, 2.7)**	**0.4 (0.2, 0.6)**	**3.3 (2.3, 4.6)**	**99.4 (98.9, 99.7)**	**33.3**
**Score ≥8**	57.8 (42.2, 72.3)	26	77.8 (76.2, 79.3)	2282	35.6	2.6 (2.0, 3.4)	0.5 (0.4, 0.8)	3.8 (2.5, 5.6)	99.2 (98.7, 99.5)	22.8
**Score ≥9**	50.0 (34.6, 65.4)	22	80.4 (79.0, 81.9)	2360	30.4	2.6 (1.9, 3.5)	0.6 (0.5, 0.8)	3.7 (2.3, 5.5)	99.1 (98.6, 99.4)	20.0
**Score ≥10**	43.2 (28.3, 59.0)	19	83.2 (81.8, 84.5)	2441	26.4	2.6 (1.8, 3.6)	0.7 (0.5, 0.9)	3.7 (2.2, 5.7)	99.0 (98.5, 99.3)	17.2
**Score ≥11**	36.4 (22.4, 52.2)	16	85.3 (83.9, 86.5)	2502	21.7	2.5 (1.7, 3.7)	0.8 (0.6, 0.9)	3.6 (2.1, 5.7)	98.9 (98.4, 99.3)	15.0
**Score ≥12**	36.4 (22.4, 52.2)	16	87.3 (86.1, 88.5)	2562	23.7	2.9 (1.9, 4.3)	0.7 (0.6, 0.9)	4.1 (2.4, 6.6)	98.9 (98.4, 99.3)	13.0
**Score ≥13**	29.5 (16.8, 45.2)	13	88.9 (87.7, 90.0)	2609	18.4	2.7 (1.7, 4.3)	0.8 (0.7, 1.0)	3.8 (2.1, 6.5)	98.8 (98.3, 99.2)	11.3
**Score ≥14**	22.7 (11.5, 37.8)	10	90.5 (89.4, 91.6)	2657	13.2	2.4 (1.4, 4.2)	0.9 (0.7, 1.0)	3.5 (1.7, 6.3)	98.7 (98.2, 99.1)	9.6
**Score ≥15**	22.7 (11.5, 37.8)	10	91.3 (90.2, 92.3)	2678	14.0	2.6 (1.5, 4.6)	0.9 (0.7, 1.0)	3.8 (1.8, 6.8)	98.7 (98.3, 99.1)	8.9

Abbreviations: LR+, positive likelihood ratio; LR-, negative likelihood ratio; PPV, positive predicted value; NPV, negative predicted value; CI, confidence interval

#### Construct Validity

The results obtained from the EFA indicated a one-factor solution. This factor explained 108.37% of the common variance (Table 3). All factor loadings were > 0.6.

The results of the CFA demonstrated a good fit of SRMR = 0.046, CFI = 0.969, and RMSEA = 0.051 (90% CI: 0.043–0.059).

Women were dichotomized as GAD or non-GAD based on the optimal cutoff score identified in our study (GAD-7 score ≥7). Women with anxiety were more likely to rate overall physical and mental health as fair and poor with *P-*value < 0.0001.

### Item Response Theory Models

#### Rasch Rating Scale Model

In the initial analysis, the thresholds of the 4 categories (0, 1, 2, and 3) did not increase monotonically. By examining the item category probability curve of the GAD-7, the threshold for “more than half the days” and “nearly every day” was lower than that of “several days” and “more than half the days”; and “more than half the days” was never the most probable response category. As proposed by Forkmann [35], we collapsed “more than half the days” and “nearly every day.” After combining, the new item category probability curve had a smooth distribution with non-descending category thresholds. Based on the principal component analysis of the residuals, the eigenvalue of the first contrast after considering the Rasch factor was 1.5, hence, the assumption of unidimensionality held for the GAD-7. The largest positive correlation was 0.07 between item 2 (“not being able to stop or control worrying”) and item 3 (“worrying too much about different things”). The assumption of local independency held as no pairs of items had correlation > 0.3. The infit mean square (MnSq) was all in the acceptable range, both before (0.82 to 1.28) and after (0.85 to 1.24) collapsing “more than half the days” and “nearly every day” (Table 5). The person separation index (PSI) for the current model was 0.75, reflecting a moderate internal consistency of the GAD-7. Before collapsing, the item difficulties in logits ranged from -0.86 (the highest level of symptomatology) for item 1 (“feeling nervous, anxious, or on edge”) to 0.64 (the lowest level of symptomatology) for item 7 (“feeling afraid as if something awful might happen”) (Table 5).

**Table 5:** Item Hierarchy and Fit Statistics for the Generalized Anxiety Disorder-7 (GAD-7) Before and After Collapsing Response Categories “Over Half the Days” and “Nearly Every Day” under the Rasch Rating Scale Model.

Item	Item Difficulty in Logits	Model SE	Infit*		Outfit*	
			MnSq	Zstd	MnSq	Zstd
**Before Collapsing**						
7. Feeling afraid as if something awful might happen	0.64	0.03	1.28	7.8	1.15	4.1
5. Being so restless that it’s hard to sit still	0.50	0.03	1.06	1.7	0.94	-1.9
4. Trouble relaxing	0.15	0.03	0.98	-0.7	0.92	-2.3
6. Becoming easily annoyed or irritable	-0.09	0.03	1.24	6.9	1.25	7.1
2. Not being able to stop or control worrying	-0.10	0.03	0.90	-3.4	0.83	-5.5
3. Worrying too much about different things	-0.24	0.03	0.82	-6.0	0.80	-6.5
1. Feeling nervous, anxious, or on edge	-0.86	0.03	0.83	-6.3	0.90	-3.0
Mean	0.00	0.03	1.01	0.0	0.97	-1.1
SD	0.46	0.00	0.17	5.3	0.15	4.6
**After Collapsing**						
7. Feeling afraid as if something awful might happen	0.74	0.04	1.24	8.3	1.25	6.7
5. Being so restless that it’s hard to sit still	0.66	0.04	0.99	-0.2	0.98	-0.6
4. Trouble relaxing	0.17	0.04	0.96	-1.4	0.97	-1.2
6. Becoming easily annoyed or irritable	-0.09	0.04	1.22	8.0	1.29	9.5
2. Not being able to stop or control worrying	-0.12	0.04	0.88	-4.8	0.87	-4.9
3. Worrying too much about different things	-0.28	0.04	0.85	-6.3	0.85	-6.0
1. Feeling nervous, anxious, or on edge	-1.07	0.04	0.89	-4.6	0.92	-2.8
Mean	0.00	0.04	1.00	-0.2	1.02	0.1
SD	0.57	0.00	0.15	5.6	0.17	5.4

Abbreviations: SE, Standard error; SD, Standard deviation; MnSq, mean square; Zstd, z-standardized

*Infit MnSq value and outfit MnSq value should range from 0.6 to 1.4, and from 0.5 to 1.7, respectively.

Table 6 shows the frequency of response categories and average ability for the GAD-7. “More than half the days” had the lowest frequency for all GAD-7 items (Table 6). The average ability for participants to endorse the 4 response categories was increasing monotonically (Table 6).

**Table 6:** Response Category Distribution and Average Ability for the Generalized Anxiety Disorder-7 (GAD-7) under the Rasch Rating Scale Model.

Item	Response category	Frequency	Percentage	Average ability	SE
1. Feeling nervous, anxious, or on edge	Not at all	763	26	-3.26	0.05
	Several days	1431	48	-1.39	0.02
	More than half the days	265	9	-0.44	0.06
	Nearly every day	519	17	1.12	0.07
2. Not being able to stop or control worrying	Not at all	1254	42	-2.71	0.04
	Several days	1185	40	-1.04	0.02
	More than half the days	211	7	0.24	0.06
	Nearly every day	328	11	1.74	0.08
3. Worrying too much about different things	Not at all	1157	39	-2.79	0.04
	Several days	1260	42	-1.10	0.02
	More than half the days	201	7	-0.04	0.06
	Nearly every day	360	12	1.72	0.07
4. Trouble relaxing	Not at all	1380	46	-2.49	0.04
	Several days	1140	38	-1.05	0.02
	More than half the days	179	6	0.35	0.08
	Nearly every day	279	9	2.01	0.08
5. Being so restless that it's hard to sit still	Not at all	1640	55	-2.30	0.04
	Several days	958	32	-0.91	0.03
	More than half the days	103	3	0.54	0.08
	Nearly every day	277	9	2.10	0.08
6. Becoming easily annoyed or irritable	Not at all	1265	42	-2.48	0.05
	Several days	1180	40	-1.17	0.03
	More than half the days	194	7	-0.14	0.09
	Nearly every day	339	11	1.59	0.09
7. Feeling afraid as if something awful might happen	Not at all	1670	56	-2.20	0.04
	Several days	953	32	-0.93	0.03
	More than half the days	138	5	0.36	0.10
	Nearly every day	217	7	2.28	0.09

Abbreviation: SE, Standard error

#### Generalized Partial Credit Model

All items discriminated well between more or less anxious women. Item 6 (“becoming easily annoyed or irritable”) had the lowest discrimination (0.97) (S2 Table). The most discriminating item was item 3 (“worrying too much about different things”) with the highest discrimination parameter of 2.05.

## Discussion

This study examined the reliability and validity of the Spanish-language version of the GAD-7 in a sample of pregnant Peruvian women assessed during early pregnancy. Among this population, the GAD-7 was a reliable measure for detecting GAD. A cutoff score of 7 or higher maximized the Youden Index which yielded a sensitivity of 73.3% (95% CI: 58.1%- 85.4%) and a specificity of 67.3% (95% CI: 65.5%- 69.0%). The results from both the exploratory and the confirmatory factor analysis confirmed the unidimentional structure of the GAD-7. Concurrent validity was supported by the extent to which higher GAD-7 scores were associated with poor self-rated physical and mental health status. The Rasch RSM further confirmed the cross-cultural validity of the GAD-7.

The reliability of the Spanish-language version of the GAD-7 was good (the Cronbach’s alpha = 0.89), agreeing with previous studies, in which the Cronbach’s alpha ranged from 0.74 [44] to 0.94 [45].

Depending on study population and language versions of the GAD-7, the recommended cutoff scores ranged from 8 to 13 [2, 17, 44–49]. In our study, to maximize the Youden Index, a cutoff score of 7 or higher yielded a sensitivity of 73% and a specificity of 67%. In a recent study of 155 pregnant Canadian women and 85 postpartum Canadian women, Simpson et al. found that that the optimal cutoff score for GAD-7 was 13 or higher with a sensitivity of 61% and a specificity of 73% [49]. Of note, their study was conducted among women who were referred for psychiatric consultation, a population that was expected to have a higher prevalence of mental disorders than women receiving prenatal care (a general obstetric population). Furthermore, other demographic and clinical characteristics of the study populations may contribute to the differences in recommended GAD cutoff scores.

In our study, at the optimal cutoff score of 7 or higher, an excellent NPV of 99% was obtained, suggesting that the GAD-7 was accurate in assuring non-GAD case status. The PPV was poor (3.3%): 3 of 100 probable cases detected by the GAD-7 actually had GAD diagnosis. The PPV depends on sensitivity, specificity, and prevalence of GAD among populations [50]. In our population, the relatively low 12-month prevalence of GAD (1.48%) based on the CIDI diagnosis might account for the low PPV, to some extent. Considering the CIDI is a fully-structured interview with strict skip patterns which does not allow the use of clinical judgment or rephrase of questions, the prevalence of the CIDI diagnosis tends to be underestimated [22, 51]. In addition, as a study conducted in clinical setting, the GAD prevalence in our study may be underestimated given the fact that respondents are known to be more comfortable admitting personal or socially unacceptable feelings and behaviors to lay interviewers in community based studies than to clinical interviewers [51–53]. Of note, in the U.S., the lifetime adulthood risk for GAD is estimated at 9% with a 12-month prevalence of 3% [1]. Globally, for expectant mothers, a prevalence of 8.5%- 10.5% has been reported [10, 54–57]. In addition, specific anxieties among pregnant women, such as anxiety about pregnancy and childbirth [6, 58–60], may have an impact on the GAD-7’s specificity and ability to adequately distinguish women who do not meet the criteria for GAD [46]. Moreover, high levels of intimate partner violence and unmet daily survival needs contribute to high levels of anxiety in the daily life of Peruvian women [61–63]. Consequently, we cannot rule out the possibility that our study participants may be less sensitive to symptoms of GAD. Future studies are needed to provide further insights into this issue.

However, relative costs and benefits of different decision thresholds also needs to be considered for screening [64]. The utility, practical values either in monetary terms or subject scales assigned to correct/incorrect screening classifications for GAD, is helpful in choosing optimal cutoff scores [48, 65]. Higher values are assigned to correct classifications and lower values for incorrect classifications. In the current study, utility is undefined. However, by maximizing the sum of sensitivity and specificity, we implicitly assumed that the utility for detecting a true positive would be 66.6 [(1–0.0148)/0.0148] times as much as the utility for detecting a true negative (where 0.0148 is the prevalence of GAD assessed by the CIDI in our population) [65, 66]. The ratio defined by the utility, if we had known, may or may not match the aforementioned ratio (66.6) defined by the Youden Index. If the ratio of utilities between the true positive and the true negative is lower than 66.6, we may want to increase the optimal cutoff score for the GAD-7 to maximize the expected utility, and vice versa. Among pregnant Peruvian women, future studies designed to assess the utility of correct classification of GAD are warranted. In addition, the availability of effective treatment for GAD should be considered in determining the optimal cutoff score of the GAD-7. There is good evidence that anxiety disorders can be effectively treated with pharmacotherapy or psychotherapy [67, 68]. Furthermore, system-based interventions coupled with screening should also be tested among pregnant women.

Using the exploratory factor analysis, confirmatory factor analysis and Rasch RSM, the results in the current study confirmed the unidimensionality of the GAD-7, which was consistent with the majority of current literature conducted among the primary care or general population [2, 14, 45]. However, in an psychiatric sample, Kertz et al. [11] failed to support the unidimentional factor structure using the confirmatory factor analysis. Item 5 (“Being so restless that it’s hard to sit still”) and item 6 (“Becoming easily annoyed or irritable”) loaded only moderately on the latent factor compared with other items. Kertz suggested that these items might also reflect a somatic tension/autonomic arousal factor. Portman et al. [69] hypothesized that there may be subtypes of GAD, including an excessive worry subtype, a somatic tension/autonomic arousal subtype, and a combined subtype [11, 69]. In our study, we observed that item 6 and item 7 (“Feeling afraid as if something awful might happen”) had the lowest loading (0.64) on the latent factor. These 2 items also had the lowest corrected item-total correlation, the highest alpha if item deleted, and the lowest discrimination. In a study that performed cultural adaption for the Spanish-language version of the GAD-7, a similar factor loading structure was observed [70]. Whether the observed low factor loadings were due to subtypes of GAD or a property of the Spanish-language version of the GAD-7 was not clear, so future exploration is required to empirically test the subtype hypothesis and validate the Spanish-language version of the GAD-7 across regions and populations in other Spanish-speaking countries.

The Spanish-language version of the GAD-7 demonstrated unidimensionality, local independence, and acceptable fit for the Rasch RSM. Following the approach suggested by Andrich and other researchers [30, 31, 35, 37, 38], we tentatively collapsed the response categories “more than half the days” and “nearly every day”, given the disordered thresholds. However, after collapsing, the model fit was not materially improved. For all 7 items, as the scores assigned to the 4 response categories increased, so did the average ability, indicating the proper order of the 4 response categories despite the disordered thresholds. The fact that few women endorsed “more than half the days” led to the disordered thresholds in numerical values [39, 71]. All items still functioned well regarding model fit. Moreover, collapsing has serious implications for the use of the GAD-7 as a screening scale because this would change the total score and original screening cutoff score and lose valuable trait information. Future study should carefully examine the reasons for disordered thresholds, and decisions in terms of collapsing should not be made solely based on disordered thresholds [39, 71].

This study has several strengths including the use of a diagnostic gold standard to assess validity, a large sample size, and an execution of a rigorous analytic plan. To our knowledge, this is the first study to examine the psychometric properties of the GAD-7 using the Rasch RSM and the GPCM. Our study expands the literature by including assessment of the Spanish-language version of the GAD-7 among pregnant Peruvian women.

Despite these strengths, this study has several limitations. Concurrent validity was examined only using self-rated health status. Data on disability measures, such as disability days, clinical visits, and the general amount of difficulty women attribute to symptoms [2, 72], were not available. In addition, the diagnostic interviews were conducted by 4 psychologists; the inter-rater reliability was not calculated. Moreover, the non-participation rate was 24.8% for participants selected for diagnostic interview which might lead to potential selection bias. Nonetheless, we observed no statistically significant difference regarding anxiety status (mean GAD-7 score) for those who completed the diagnostic interview and those who did not (mean = 6.0, SD = 5.5 vs. mean = 5.8, SD = 5.0, *P*-value = 0.84). Furthermore, current data were cross-sectional collected during early pregnancy. As anxiety levels might vary during the course of pregnancy, longitudinal studies are warranted to help understand how GAD symptom severity changes across pregnancy trimesters.

In conclusion, our results suggest that the Spanish-language version of the GAD-7 may be used as a screening tool for pregnant women. The GAD-7 has good reliability, factorial validity, and concurrent validity. In this population, the optimal cutoff score obtained by maximizing the Youden Index (GAD-7 score ≥ 7) should be considered cautiously; women who screened positive may require further investigation to confirm GAD diagnosis. Future studies that evaluate the utility of correct classification and tests the effectiveness of current GAD treatments would provide more evidence for determining the optimal cutoff score for pregnant women.

## Supporting Information

S1 Table Begg and Greens Adjusted Sensitivity and Specificity for Generalized Anxiety Disorder Diagnosis across Various Cutoff Scores of the Generalized Anxiety Disorder-7 (GAD-7). (DOCX)

S2 Table Estimated Item Discrimination and Category Intersection Parameters of the Generalized Anxiety Disorder-7 (GAD-7) Using the Generalized Partial Credit Model (GPCM). (DOCX)
