# Supplementary material for: Diagnostic Validity of the Generalized Anxiety Disorder - 7 (GAD-7) among Pregnant Women
Source: PLoS One. 2015 Apr 27;10(4):e0125096. doi: 10.1371/journal.pone.0125096 (PMC4411061; doi:10.1371/journal.pone.0125096)
Supplement: S1 Table — (DOCX) [file pone.0125096.s001.docx]

**Table S1. Begg and Greens Adjusted Sensitivity and Specificity for Generalized Anxiety Disorder Diagnosis across Various Cutoff Scores of the Generalized Anxiety Disorder-7 (GAD-7)**

| **Cutoff Scores** | **Sensitivity**  **(95%CI)** | **# True**  **positive** | **Specificity**  **(95%CI)** | **# True negative** | | **Youden**  **index** | **LR+**  **(95% CI)** | **LR-**  **(95%CI)** | **PPV**  **(95%CI)** | **NPV**  **(95%CI)** | **Prevalence** |
| --- | --- | --- | --- | --- | --- | --- | --- | --- | --- | --- | --- |
| **Score ≥1** | 100.0 (92.0, 100.0) | 44 | 8.8 (7.8, 9.8) | | 257 | 8.8 | 1.1 (1.1, 1.1) |  | 1.6 (1.2, 2.2) | 100.0 (98.6, 100.0) | 91.3 |
| **Score ≥2** | 100.0 (92.1, 100.0) | 44 | 17.5 (16.1, 18.9) | | 513 | 17.5 | 1.2 (1.2, 1.2) |  | 1.8 (1.3, 2.4) | 100.0 (99.3, 100.0) | 82.8 |
| **Score ≥3** | 93.3 (81.7, 98.6) | 42 | 27.2 (25.6, 28.9) | | 799 | 20.5 | 1.3 (1.2, 1.4) | 0.3 (0.1, 0.7) | 1.9 (1.4, 2.6) | 99.6 (98.9, 99.9) | 73.1 |
| **Score ≥4** | 93.3 (81.7, 98.6) | 42 | 36.8 (35.0, 38.6) | | 1080 | 30.1 | 1.5 (1.4, 1.6) | 0.2 (0.1, 0.5) | 2.2 (1.6, 3.0) | 99.7 (99.2, 99.9) | 63.7 |
| **Score ≥5** | 93.3 (81.7, 98.6) | 42 | 46.3 (44.5, 48.2) | | 1375 | 39.6 | 1.7 (1.6, 1.9) | 0.1 (0.1, 0.4) | 2.6 (1.9, 3.5) | 99.8 (99.4, 100.0) | 53.8 |
| **Score ≥6** | 73.3 (58.1, 85.4) | 33 | 56.1 (54.3, 57.9) | | 1646 | 29.4 | 1.7 (1.4, 2.0) | 0.5 (0.3, 0.8) | 2.5 (1.7, 3.5) | 99.3 (98.7, 99.6) | 44.4 |
| **Score ≥7** | 73.3 (58.1, 85.4) | 32 | 67.3 (65.5, 69.0) | | 1974 | 40.6 | 2.2 (1.9, 2.7) | 0.4 (0.2, 0.6) | 3.3 (2.3, 4.6) | 99.4 (98.9, 99.7) | 33.3 |
| **Score ≥8** | 57.8 (42.2, 72.3) | 26 | 77.8 (76.2, 79.3) | | 2282 | 35.6 | 2.6 (2.0, 3.4) | 0.5 (0.4, 0.8) | 3.8 (2.5, 5.6) | 99.2 (98.7, 99.5) | 22.8 |
| **Score ≥9** | 50.0 (34.6, 65.4) | 22 | 80.4 (79.0, 81.9) | | 2360 | 30.4 | 2.6 (1.9, 3.5) | 0.6 (0.5, 0.8) | 3.7 (2.3, 5.5) | 99.1 (98.6, 99.4) | 20.0 |
| **Score ≥10** | 43.2 (28.3, 59.0) | 19 | 83.2 (81.8, 84.5) | | 2441 | 26.4 | 2.6 (1.8, 3.6) | 0.7 (0.5, 0.9) | 3.7 (2.2, 5.7) | 99.0 (98.5, 99.3) | 17.2 |
| **Score ≥11** | 36.4 (22.4, 52.2) | 16 | 85.3 (83.9, 86.5) | | 2502 | 21.7 | 2.5 (1.7, 3.7) | 0.8 (0.6, 0.9) | 3.6 (2.1, 5.7) | 98.9 (98.4, 99.3) | 15.0 |
| **Score ≥12** | 36.4 (22.4, 52.2) | 16 | 87.3 (86.1, 88.5) | | 2562 | 23.7 | 2.9 (1.9, 4.3) | 0.7 (0.6, 0.9) | 4.1 (2.4, 6.6) | 98.9 (98.4, 99.3) | 13.0 |
| **Score ≥13** | 29.5 (16.8, 45.2) | 13 | 88.9 (87.7, 90.0) | | 2609 | 18.4 | 2.7 (1.7, 4.3) | 0.8 (0.7, 1.0) | 3.8 (2.1, 6.5) | 98.8 (98.3, 99.2) | 11.3 |
| **Score ≥14** | 22.7 (11.5, 37.8) | 10 | 90.5 (89.4, 91.6) | | 2657 | 13.2 | 2.4 (1.4, 4.2) | 0.9 (0.7, 1.0) | 3.5 (1.7, 6.3) | 98.7 (98.2, 99.1) | 9.6 |
| **Score ≥15** | 22.7 (11.5, 37.8) | 10 | 91.3 (90.2, 92.3) | | 2678 | 14.0 | 2.6 (1.5, 4.6) | 0.9 (0.7, 1.0) | 3.8 (1.8, 6.8) | 98.7 (98.3, 99.1) | 8.9 |
| **Score ≥16** | 24.4 (12.9, 39.5) | 10 | 92.1 (91.0, 93.0) | | 2701 | 16.5 | 3.1 (1.8, 5.2) | 0.8 (0.7, 1.0) | 4.5 (2.3, 7.9) | 98.8 (98.3, 99.1) | 8.2 |
| **Score ≥17** | 22.7 (11.5, 37.8) | 10 | 93.6 (92.6, 94.4) | | 2746 | 16.3 | 3.5 (2.0, 6.2) | 0.8 (0.7, 1.0) | 5.1 (2.4, 9.1) | 98.8 (98.3, 99.2) | 6.6 |
| **Score ≥18** | 22.7 (11.5, 37.8) | 10 | 94.2 (93.3, 95.1) | | 2765 | 16.9 | 3.9 (2.2, 6.9) | 0.8 (0.7, 1.0) | 5.6 (2.7, 10.0) | 98.8 (98.3, 99.2) | 6.0 |
| **Score ≥19** | 9.1 (2.5, 21.7) | 4 | 95.6 (94.8, 96.3) | | 2804 | 4.7 | 2.1 (0.8, 5.3) | 1.0 (0.9, 1.0) | 3.0 (0.8, 7.5) | 98.6 (98.1, 99.0) | 4.5 |
| **Score ≥20** | 8.9 (2.5, 21.2) | 4 | 96.3 (95.6, 97.0) | | 2826 | 5.2 | 2.4 (0.9, 6.3) | 1.0 (0.9, 1.0) | 3.6 (1.0, 8.9) | 98.6 (98.1, 99.0) | 3.8 |
| **Score ≥21** | 0.0 (0.0, 8.0) | 0 | 97.0 (96.3, 97.6) | | 2846 | -0.3 |  | 1.0 (1.0, 1.0) | 0.0 (0.0, 4.1) | 98.5 (98.0, 98.9) | 3.0 |

LR+: positive likelihood ratio; LR-: negative likelihood ratio; PPV: positive predicted value; NPV: negative predicted value; CI: confidence interval.
