# Supplementary material for: Diagnostic Validity of the Generalized Anxiety Disorder - 7 (GAD-7) among Pregnant Women
Source: PLoS One. 2015 Apr 27;10(4):e0125096. doi: 10.1371/journal.pone.0125096 (PMC4411061; doi:10.1371/journal.pone.0125096)
Supplement: S2 Table — (DOCX) [file pone.0125096.s002.docx]

**Table S2. Estimated Item Discrimination and Category Intersection Parameters of the Generalized Anxiety Disorder-7 (GAD-7) Using the Generalized Partial Credit Model (GPCM)**

| **Items** | **Discrimination**  **parameter** | |  | **Category intersection parameters** | | | | | | | |
| --- | --- | --- | --- | --- | --- | --- | --- | --- | --- | --- | --- |
|  |  |  |  | **Category 0 and 1^*^** | |  | **Category 1and 2^*^** | |  | **Category 2and 3^*^** | |
|  | **Estimates** | **SE** |  | **Estimates** | **SE** |  | **Estimates** | **SE** |  | **Estimates** | **SE** |
| 1. Feeling nervous, anxious, or on edge | 1.58 | 0.07 |  | -0.84 | 0.04 |  | 1.35 | 0.07 |  | 0.50 | 0.06 |
| 2. Not being able to stop or control worrying | 2.04 | 0.09 |  | -0.17 | 0.03 |  | 1.39 | 0.06 |  | 1.02 | 0.06 |
| 3. Worrying too much about different things | 2.05 | 0.09 |  | -0.28 | 0.03 |  | 1.40 | 0.06 |  | 0.90 | 0.06 |
| 4. Trouble relaxing | 1.46 | 0.07 |  | -0.01 | 0.04 |  | 1.83 | 0.08 |  | 0.95 | 0.08 |
| 5. Being so restless that it's hard to sit still | 1.50 | 0.07 |  | 0.31 | 0.04 |  | 2.14 | 0.10 |  | 0.67 | 0.09 |
| 6. Becoming easily annoyed or irritable | 0.97 | 0.05 |  | -0.11 | 0.05 |  | 2.28 | 0.12 |  | 0.46 | 0.11 |
| 7. Feeling afraid as if something awful might happen | 1.09 | 0.05 |  | 0.48 | 0.05 |  | 2.39 | 0.12 |  | 0.89 | 0.11 |

SE: Standard error

**^*^**Category 0: “Not at all”; category 1: “Several days”; category 2: “More than half the days”; category 3 “Nearly every day”
